# Long-term efficacy of oral calcium polystyrene sulfonate for hyperkalemia in CKD patients

**DOI:** 10.1371/journal.pone.0173542

**Published:** 2017-03-22

**Authors:** Mi-Yeon Yu, Jee Hyun Yeo, Joon-Sung Park, Chang Hwa Lee, Gheun-Ho Kim

**Affiliations:** Department of Internal Medicine, Hanyang University College of Medicine, Seoul, South Korea; The University of Tokyo, JAPAN

## Abstract

**Background:**

Calcium polystyrene sulfonate (CPS) has long been used to treat hyperkalemia in patients with chronic kidney disease (CKD). However, its efficacy and safety profile have not been systematically explored. We investigated the long-term efficacy of oral CPS for treating mild hyperkalemia on an outpatient basis.

**Methods:**

We performed a retrospective analysis of ambulatory CKD patients who were prescribed CPS for > 1 week because of elevated serum potassium levels > 5.0 mmol/L. Patients were divided into four groups according to the length of time that they took a fixed dosage of CPS (Group 1, < 3 months; Group 2, 3–6 months; Group 3, 6–12 months; and Group 4, > 1 year). Response was defined as a decrease in the serum potassium level (> 0.3 mmol/L) after treatment with CPS.

**Results:**

We enrolled a total of 247 adult patients with a basal eGFR level of 30 ± 15 mL/min/1.73 m^2^. All patients took small doses of CPS (8.0 ± 3.6 g/d), and serum potassium decreased in a dose-dependent fashion. Serum potassium of all patients decreased significantly from 5.8 ± 0.3 mmol/L to 4.9 ± 0.7 mmol/L with CPS treatment (*P* < 0.001). The response rates were 79.9%, 71.4%, 66.7%, and 86.8% in Groups 1, 2, 3, and 4, respectively. No serious adverse effects were reported during CPS administration, though constipation was noted in 19 patients (8%).

**Conclusion:**

Small doses of oral CPS are effective and safe for controlling mild hyperkalemia in CKD patients over a long period of time.

## Introduction

Hyperkalemia is an important complication of chronic kidney disease (CKD) because urinary potassium excretion gradually decreases with declining glomerular filtration rate (GFR) [1]. Apart from glomerular filtration, tubular secretion of potassium occurring in the cortical collecting duct is the primary determinant of urinary potassium excretion. Thus, hyporeninemic hypoaldosteronism and angiotensin converting enzyme inhibitor (ACEI) or angiotensin II receptor blockade (ARB) therapy increase the risk of hyperkalemia in CKD patients [2]. This is the major obstacle to the use of ACEIs and ARBs as renoprotective agents. In particular, patients with diabetic kidney disease may benefit from potassium lowering agents because hyperkalemia is difficult to be avoided by dietary potassium restriction alone. Contradictorily, the typical healthy diabetic diet is often rich in potassium. Hyperosmolality, acidosis, insulin deficiency, and medications are all contributory to the transcellular shift of potassium in diabetic patients. Furthermore, hypoaldosteronism is frequently induced by renin hyposecretion or renin-angiotensin system blockades [3].

Treatment options for hyperkalemia are well documented [4], and urgent therapy is indicated for rapid and substantial elevations in serum potassium. However, less aggressive therapy to remove potassium may be recommended for patients with modest elevations in serum potassium without cardiac and neuromuscular manifestations. For this purpose, cation exchange resins have been used in clinical practice.

Sodium polystyrene sulfonate (SPS) was previously used together with a cathartic agent to treat acute hyperkalemia in patients with end stage renal disease (ESRD). However, it is seldom used because of a poor side-effect profile and uncertain efficacy [5]. Recently, new agents such as patiromer and sodium zirconium cyclosilicate have emerged for the treatment of hyperkalemia. However, they are unavailable in many countries, despite promising results from randomized controlled trials [6–9].

In contrast, calcium polystyrene sulfonate (CPS) has long been used for patients with advanced CKD in many parts of the world. It entraps potassium in the distal colon in exchange for calcium. This may have an advantage over SPS because it avoids sodium retention and supplements calcium. However, few clinical studies have evaluated the usefulness of CPS in the treatment of hyperkalemia. This study was undertaken to investigate the long-term efficacy of oral CPS for treating mild hyperkalemia on an outpatient basis.

## Materials and methods

### Patients

A retrospective analysis was done using electronic medical records. We enrolled adult (> 18 years old) patients who visited our outpatient department due to CKD between January 2010 and December 2014. All patients took oral CPS for > 1 week due to elevated serum potassium levels > 5.0 mmol/L. According to patient preference, we used two different formulae of CPS: Kalimate^®^ granules (Kunwha Pharmaceutical, Seoul, Korea) and Argamate^®^ jelly (JW Pharmaceutical, Seoul, Korea). A single dose of each formula had 5 g of CPS.

**Fig 1:**
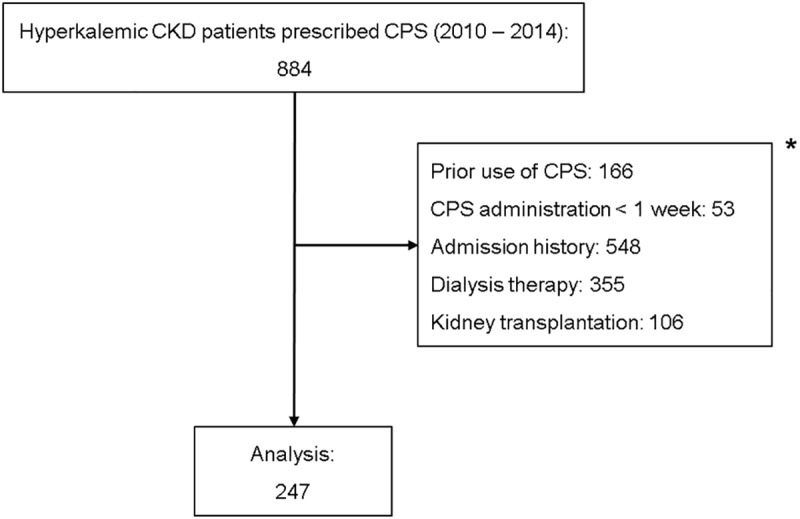
Patient enrollment. *Patient numbers among categories overlapped.

Eight hundred eighty-four patients were initially screened, but 247 patients were finally analyzed because we excluded those with prior CPS use, administration for less than a week, admission history, dialysis therapy, and kidney transplantation (Fig 1). This study was approved by the review board of Hanyang University Hospital (IRB File No. 2016-12-009).

### Data collection

In addition to demographic parameters, we collected basal laboratory data before CPS treatment, including complete blood cell count (CBC), serum electrolytes, blood urea nitrogen (BUN) and serum creatinine. Blood samples were collected at fasting states in the morning. The follow-up laboratory data including serum potassium level was determined at the scheduled regular visits. Estimated glomerular filtration rate (eGFR) was calculated using the Chronic Kidney Disease Epidemiology (CKD-EPI) collaboration equation [10]. Response was defined as a decrease in the serum K^+^ by > 0.3 mmol/L after treatment with CPS, and characteristics were compared between responders and non-responders. The criterion of the response was based upon the results of placebo groups in the recent clinical trials [6–9]. We divided patients into four groups based on the duration of CPS medication: Group 1, less than 3 months; Group 2, 3 to 6 months; Group 3, 6 months to 1 year; and Group 4, 1 year or more. Only the periods with a fixed dosage of CPS were evaluated, although patients may have had varied doses over time. Throughout the follow-up period, we searched for any adverse events associated with CPS medication. Constipation was assessed based upon patient complaints or prescription of laxatives.

#### Statistical analysis

Continuous data were described as means ± standard deviation. Statistical comparisons between two groups were performed using the paired and unpaired t-test where appropriate. When analyses involved data from ≥ 3 groups, the one-way analysis of variance (ANOVA) test was used for comparison. Categorical data were expressed as frequency (and proportion), and the association between variables was analyzed using contingency tables and the chi-square test. Logistic regression analysis was used to evaluate associations between parameters and responses to CPS. A two-tailed *P* < 0.05 was considered statistically significant. All statistical analyses were performed using StatView 4.01 (SAS Institute Inc., Cary, NC).

## Results

### Baseline patient characteristics

**Fig 2:**
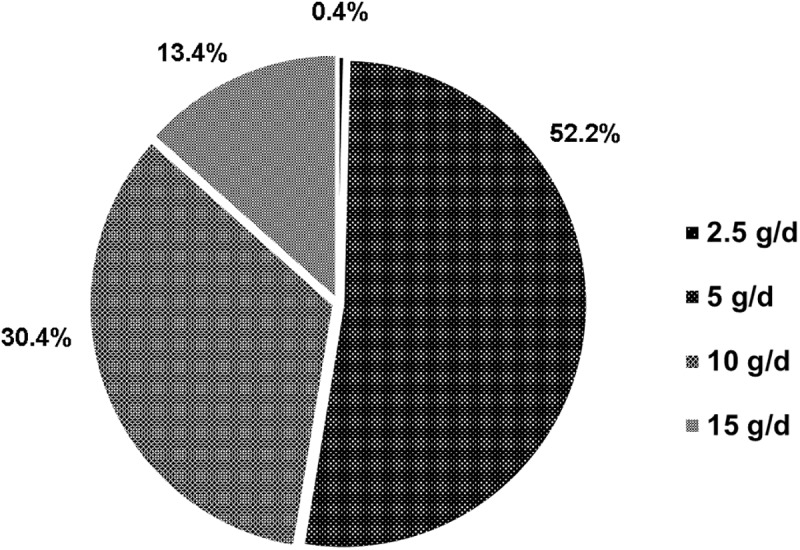
Dosage distribution for the use of calcium polystyrene sulfonate.

A total of 247 patients (CKD stage 2, 8; stage 3, 105; stage 4, 92; and stage 5, 42) were enrolled, and Table 1 summarizes baseline characteristics. Kalimate^®^ (granule form) was more frequently taken than Argamate^®^ jelly. All patients used small doses of CPS, ranging from 2.5 to 15 g per day (Fig 2). The mean daily dose of CPS was 8 g, and the mean medication duration was 5.6 + 8.7 months.

**Table 1:** Patient characteristics at baseline.

Variable	Total (n = 247)
Age (years)	64 ± 14
Male	136 (55.1%)
Kalimate^®^	169 (68.4%)
Daily dose of CPS (g)	8.0 ± 3.6
Medication duration (months)	5.6 ± 8.7
Causes of CKD	
Diabetic kidney disease	110 (44.5%)
Hypertensive nephrosclerosis	55 (22.3%)
Chronic glomerulonephritis	35 (14.2%)
Polycystic kidney disease	4 (1.6%)
ACEI or ARB use	155 (62.8%)
Hemoglobin (g/dL)	10.7 ± 1.8
BUN (mg/dL)	46 ± 22
Serum creatinine (mg/dL)	2.8 ± 1.8
eGFR (mL/min/1.73 m^2^)	30 ± 15
Serum sodium (mmol/L)	140 ± 3
Serum potassium (mmol/L)	5.8 ± 0.3

Values are expressed as mean ± standard deviation for continuous variables and number (%) for categorical variables.

CPS, calcium polystyrene sulfonate; CKD, chronic kidney disease; ACEI, angiotensin converting enzyme inhibitor; ARB, angiotensin II receptor blockade; BUN, blood urea nitrogen; eGFR, estimated glomerular filtration rate.

Diabetes mellitus was the most common cause of CKD (44.5%). The mean eGFR was 30 mL/min/1.73 m^2^, but ACEIs or ARBs were frequently used (Table 1). The basal serum potassium was 5.8 ± 0.3 mmol/L.

Table 2 shows that hemoglobin, blood urea nitrogen, and serum creatinine significantly changed according to CKD stage (*P* < 0.001). In patients with hyperkalemia, however, serum sodium and potassium did not differ significantly between stages.

**Table 2:** Laboratory data according to CKD stage.

	Stage 2	Stage 3	Stage 4	Stage 5	P*
	(n = 8)	(n = 105)	(n = 92)	(n = 42)	
Hemoglobin (g/dL)	12.6 ± 1.8	11.3 ± 1.7	10.4 ± 1.6	9.7 ± 1.4	<0.001
BUN (mg/dL)	21.7 ± 9.1	32.1 ± 10.0	49.7 ± 14.7	76.8 ± 20.3	<0.001
Creatinine (mg/dL)	1.1 ± 0.1	1.6 ± 0.3	2.9 ± 0.6	6.0 ± 2.1	<0.001
Sodium (mmol/L)	139.5 ± 3.2	139.9 ± 2.9	139.8 ± 2.7	140.1 ± 3.3	0.952
Potassium (mmol/L)	5.7 ± 0.4	5.7 ± 0.3	5.8 ± 0.3	5.8 ± 0.4	0.598
Total CO_2_ (mmol/L)	27.3 ± 3.4	24.5 ± 3.7	22.6 ± 3.5	20.0 ± 3.4	0.002

Values are expressed as mean ± standard deviation.

BUN, blood urea nitrogen.

* Calculated with the one-way ANOVA test.

### Treatment efficacy

**Fig 3:**
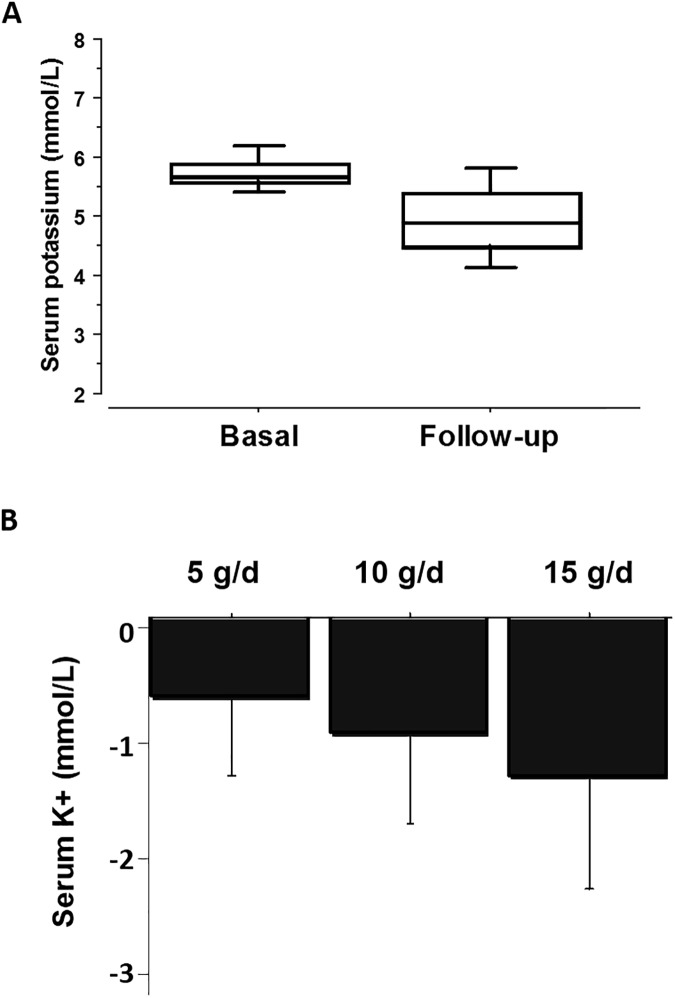
Effects of calcium polystyrene sulfonate on serum potassium. A: Serum potassium concentrations were compared before and after administration of calcium polystyrene sulfonate (*, *P* < 0.001 by paried t-test). B: Serum potassium concentrations were lowered by calcium polystyrene sulfonate in a dose-dependent fashion (*P* < 0.001 by one-way ANOVA test).

When all patients were taken together, CPS treatment significantly decreased the serum potassium level from 5.8 ± 0.3 mmol/L to 4.9 ± 0.7 mmol/L (*P* < 0.001, Fig 3A). In other words, hyperkalemia was corrected to below 5.0 mmol/L in 57.5% of our patients. As shown in Fig 3B, the serum potassium-lowering effect was dose-dependent.

When patients were divided into four groups according to the medication duration, all groups had significant decreases in serum potassium (Table 3). The response rate and the decrease in serum potassium were similar between groups, ranging from 66.7 to 86.8%.

**Table 3:** Results from different groups according to the duration of medication administration.

	Group 1	Group 2	Group 3	Group 4
	(n = 144)	(n = 35)	(n = 30)	(n = 38)
Basal serum K^+^ (mmol/L)	5.8 ± 0.4	5.7 ± 0.4	5.7 ± 0.2	5.7 ± 0.3
Follow-up serum K^+^ (mmol/L)	4.9 ± 0.7*	4.9 ± 0.7*	5.1 ± 0.6*	4.7 ± 0.6*
Daily CPS dose (g/day)	8.2 ± 3.6	7.2 ± 3.6	8.7 ± 3.5	7.6 ± 3.6
Medication duration (months)	1.3 ± 0.8	4.6 ± 0.8	9.2 ± 1.7	23.1 ± 10.5
Response (%)	79.9	71.4	66.7	86.8

Values are expressed as mean ± standard deviation.

CPS, calcium polystyrene sulfonate.

*P < 0.001 *versus* basal serum K^+^, comparison with the paired t-test.

Next, we searched for factors that inhibited the effect of CPS. Age, eGFR and daily CPS dose did not differ between responders and non-responders. As expected, responders used a larger dose of CPS than non-responders. The serum potassium-lowering effect was more easily induced when the serum potassium level was higher (Table 4). The response to CPS was not affected by sex, CKD stage, and CPS formulae (Table 5). However, ACEI or ARB users were significantly less responsive to CPS than non-users (74 vs. 86%, *P* < 0.05). Interestingly, the response in ACEI or ARB users was not significantly affected by discontinuation of ACEIs or ARBs (*P* = 0.676). Table 6 shows the results of logistic regression analysis to determine the predictive factors for the response to the CPS treatment. Multivariable logistic regression analysis revealed that basal serum potassium [odds ratio (OR) 3.649; 95% *CI* (confidence interval) 1.217–10.941, *P* = 0.021] and use of ACEi/ARB (OR 0.456; 95% *CI* 0.226–0.919, *P* = 0.028) were independently associated with the response to the CPS treatment.

**Table 4:** Comparison of parameters between responders and non-responders.

	Responder	Non-responder	P*
	(n = 193)	(n = 54)	
Age (year)	63.6 ± 14.4	63.9 ± 13.6	0.890
eGFR (mL/min/1.73 m^2^)	30.2 ± 15.2	28.4 ± 16.2	0.466
Basal serum potassium (mmol/L)	5.8 ± 0.4	5.7 ± 0.3	0.005
Daily CPS dose (g/d)	8.3 ± 3.6	7.2 ± 3.2	0.048

Values are expressed as mean ± standard deviation.

eGFR, estimated glomerular filtration rate.

*Comparisons were made with the unpaired t-test test.

**Table 5:** Association between patient characteristics and response to CPS.

Variable	N	Response (%)	P*
**Sex**			
Male	101	74	0.103
Female	92	83	
**CKD stage**			
Stage 2	6	75	0.658
Stage 3	86	82	
Stage 4	70	76	
Stage 5	31	74	
**CPS formula**			
Kalimate^®^	139	81	0.123
Argamate^®^	54	72	
**ACEI/ARB**			
Use	114	74	0.024
No use	79	86	

CKD, chronic kidney disease; CPS, calcium polystyrene sulfonate; ACEI, angiotensin converting enzyme inhibitor; ARB, angiotensin II receptor blockade.

*Chi-square tests were used to compare dichotomous variables.

**Table 6:** Results of logistic regression analysis for responses to CPS.

Variable	Univariate		Multivariate*	
	OR (95% CI)	*P*	OR (95% CI)	*P*
Age (year)	0.998 (0.977–1.020)	0.889		
Sex (male)	0.596 (0.319–1.114)	0.105		
CKD stage		0.661		
Stage 2	Reference			
Stage 3	1.509 (0.282–8.060)	0.630		
Stage 4	1.061 (0.200–5.637)	0.945		
Stage 5	0.939 (0.165–5.362)	0.944		
eGFR (mL/min/1.73 m^2^)	1.007 (0.988–1.028)	0.465		
Basal serum potassium (mmol/L)	4.048 (1.499–10.936)	0.006	3.649 (1.217–10.941)	0.021
CPS formula (Kalimate^®^)	1.638 (0.872–3.079)	0.125		
Daily CPS dose (g/d)	1.594 (1.000–2.540)	0.050	1.228 (0.742–2.031)	0.424
ACEI/ARB	0.458 (0.230–0.909)	0.026	0.456 (0.226–0.919)	0.028

OR, odds ratio; CI, confidence interval; CKD, chronic kidney disease; eGFR, estimated glomerular filtration rate; CPS, calcium polystyrene sulfonate; ACEI, angiotensin converting enzyme inhibitor; ARB, angiotensin II receptor blockade.

*Logistic regression analyses entering variables with *P* < 0.05 in univariate analysis.

### Adverse events

Many patients complained of an unpleasant taste when taking CPS. Constipation was noted in 19 patients (7.7%). However, no serious adverse events were reported, including colonic necrosis.

## Discussion

Our results indicate that CPS is an effective agent for the control of mild hyperkalemia in CKD patients. In particular, it can be chronically used in outpatients without serious adverse effects. Although most patients with CKD appear to tolerate serum potassium levels of 5.0 to 5.5mmol/L with no significant clinical manifestations, serum potassium levels >5.0 mmol/L are associated with increased mortality in patients with stage 3–4 CKD [11].

At present, the optimal treatment of asymptomatic chronic hyperkalemia remains unclear. As a cation exchange resin, polystyrene sulfonate is supplied either in the sodium or calcium form. SPS (Kayexalate^®^), first introduced in the 1950s, exchanges sodium for calcium, ammonium, and magnesium in addition to potassium [12]. Like patiromer, CPS binds potassium in exchange for calcium. Thus, it can avoid the sodium retention that might be induced by SPS. On the other hand, ZS-9 (sodium zirconium cyclosilicate) is a highly selective sorbent that entraps potassium in the intestinal tract in exchange for sodium and hydrogen [6]. Two new agents, ZS-9 and patiromer, were recently highlighted in randomized controlled clinical trials, providing evidence for their efficacy and safety [6–9]. On the other hand, the results of SPS use were unsatisfactory [4]. Few clinical studies have documented the efficacy of long-term CPS use, although occasional case reports on colonic necrosis have been published.

SPS has been administered with sorbitol to avoid bowel obstruction and to facilitate rapid delivery to the distal colon, where potassium binding is most effective [13]. The initial placebo-controlled trial failed to demonstrate a decrease in serum potassium within 12 hours of oral ingestion in ESRD patients [5]. However, a later double-blind, randomized, placebo-controlled trial conducted in 33 ambulatory patients with CKD and mild hyperkalemia (5.0–5.9 mmol/L) indicated that serum potassium was significantly reduced by 30 g/day of Kayexalate without sorbitol for 7 days [14]. There has been only one report on the long-term use of SPS, in which 14 patients were treated for a median time of 14.5 months [15]. Gastrointestinal adverse effects of SPS are common, including loss of appetite, nausea, vomiting, and constipation. Colonic necrosis is rarely associated, but may be fatal [16].

Because of the warning issued by the U.S. Food and Drug Administration to avoid administration of Kayexalate with sorbitol [17], our patients were given low doses of CPS without sorbitol and with a small amount of water. The majority of our CKD patients were in stages 3 and 4, and small numbers of patients with CKD stage 2 and 5 were included.

We found that over varied periods, serum potassium concentration was effectively lowered by small doses of CPS. The effect was not different between the two formulae. As expected, there was a dose-response relationship between CPS dose (5 to 15 g/d) and serum potassium decrease. Serum potassium concentrations decreased by 0.68 mmol/L at 5 g/d, 0.96 mmol/L at 10 g/d, and 1.32 mmol/L at 15 g/d. These responses were similar to the results from Tomino et al., in which Argamate jelly was used in 23 CKD patients [18].

Data on the long-term use of SPS are sparse. In this respect, our study is important because 247 patients were given CPS at a fixed dosage for up to 56 months. In particular, the serum potassium decrease held steady over four different periods. Thus, drug resistance or tolerance was not induced despite a long duration of medication administration. In other words, there were no notable patient compliance or adherence issues.

In most of our patients, CPS was useful to treat hyperkalemia. Its response was not affected by age, sex, CKD stage, or CPS formulae. ACEIs and ARBs are frequently used in CKD patients to reduce proteinuria and retard progression to ESRD [19]. In total, 62.8% of our patients used ACEIs or ARBs; as expected, the serum potassium-lowering effect of CPS was slightly reduced by preexisting use of ACEIs or ARBs. On the other hand, in the group of 155 patients who used ACEIs/ARBs, the effects of CPS were not affected by discontinuation of ACEIs or ARBs. Thus, we demonstrated that low doses of CPS were also effective in CKD patients using ACEIs/ARBs and suggest that ACEIs/ARBs might be continued as long as they are combined with effective doses of CPS. Consistent with these results, Chernin et al. reported that SPS was effective as a secondary preventive measure for hyperkalemia induced by ACEIs/ARBs in CKD patients with heart disease [15].

Finally, we examined adverse events from medical records. Although CPS was frequently unpalatable, it did not produce serious gastrointestinal disturbances. No episodes of colonic necrosis or perforation were noted. Constipation was an important adverse effect, but was easily relieved by laxatives. The frequency of constipation was not high and appeared comparable to that of new agents, such as ZS-9 and patiromer [6, 7].

There have been quite a few case reports of colonic necrosis in patients using CPS [20–26]. In some cases, the CPS dosage was described; daily doses ranged from 45 to 90 g/d [26] and were given with sorbitol [25] or high-dose hydrocortisone [20, 24]. Based on this information, we suspect that low doses of CPS given alone by mouth may be safe. On the other hand, it is unclear whether this complication is independently associated with CPS. A retrospective cohort study revealed no significant association between SPS and colonic necrosis [27].

This study has several limitations. Our analysis was retrospective in nature, and some measurements including serum calcium and phosphorus were not included. We had no placebo controls, but a reasonable response criterion (serum potassium lowering > 0.3 mmol/L) was applied according to the results of recent clinical trials [6–9]. There is a possibility that we underestimated the incidence of constipation. We counted only medication periods of a fixed dosage, but this would not create a bias. Although this study enrolled a small number of patients, no previous studies on CPS enrolled more patients or followed them for longer periods than we have.

In conclusion, we have demonstrated that low doses of oral CPS are effective and safe for treating mild hyperkalemia in ambulatory CKD patients over a long period of time. This might be helpful for maintaining ACEIs/ARBs in patients with CKD. Because emerging agents for potassium binding or entrapping may be safer, head-to-head-trials are necessary to test which is most cost-effective in treating mild hyperkalemia in CKD patients.

## Supporting information

S1 File Individual patient information. (XLSX)
